# The effect of sleep–wake intraindividual variability in digital cognitive behavioral therapy for insomnia: a mediation analysis of a large-scale RCT

**DOI:** 10.1093/sleep/zsab118

**Published:** 2021-05-08

**Authors:** Cecilie L Vestergaard, Øystein Vedaa, Melanie R Simpson, Patrick Faaland, Daniel Vethe, Kaia Kjørstad, Knut Langsrud, Lee M Ritterband, Børge Sivertsen, Tore C Stiles, Jan Scott, Håvard Kallestad

**Affiliations:** 1 Department of Mental Health, Norwegian University of Science and Technology, Trondheim, Norway; 2 St. Olavs University Hospital, Østmarka, Trondheim, Norway; 3 Department of Health Promotion, Norwegian Institute of Public Health, Bergen, Norway; 4 Voss District Psychiatric Hospital, NKS Bjørkeli, Voss, Norway; 5 Department of Public Health and Nursing, Norwegian University of Science and Technology, Trondheim, Norway; 6 Clinical Research Unit Central Norway, St. Olavs Hospital, Trondheim, Norway; 7 Center for Behavioral Health and Technology, Department of Psychiatry and Neurobehavioral Sciences, University of Virginia, Charlottesville, VA, USA; 8 Department of Research & Innovation, Helse-Fonna HF, Haugesund, Norway; 9 Department of Psychology, Norwegian University of Science and Technology, Norway; 10 University of Newcastle, Newcastle, United Kingdom

**Keywords:** mediation analysis, CBT-I, sleep–wake cycle, intraindividual variability, randomized controlled trial

## Abstract

**Study Objectives:**

Digital cognitive behavioral therapy for insomnia (dCBT-I) is an effective treatment for insomnia. However, less is known about mediators of its benefits. The aim of the present study was to test if intraindividual variability in sleep (IIV) was reduced with dCBT-I, and whether any identified reduction was a mediator of dCBT-I on insomnia severity and psychological distress.

**Methods:**

In a two-arm randomized controlled trial (RCT), 1720 adults with insomnia (dCBT-I = 867; patient education about sleep = 853) completed the Insomnia Severity Index (ISI), the Hospital Anxiety and Depression Scale (HADS) and sleep diaries, at baseline and 9-week follow-up. Changes in IIV were analyzed using linear mixed modeling followed by mediation analyses of ISI, HADS, and IIV in singular sleep metrics and composite measures (behavioral indices (BI-Z) and sleep disturbance indices (SI-Z)).

**Results:**

dCBT-I was associated with reduced IIV across all singular sleep metrics, with the largest between-group effect sizes observed for sleep onset latency (SOL). Reduced IIV for SOL and wake after sleep onset had the overall greatest singular mediating effect. For composite measures, SI-Z mediated change in ISI (*b* = −0.74; 95% confidence interval (CI) −1.04 to −0.52; 13.3%) and HADS (*b* = −0.40; 95% CI −0.73 to −0.18; 29.2%), while BI-Z mediated minor changes.

**Conclusion:**

Reductions in IIV in key sleep metrics mediate significant changes in insomnia severity and especially psychological distress when using dCBT-I. These findings offer important evidence regarding the therapeutic action of dCBT-I and may guide the future development of this intervention.

**Clinical trials:**

*Name:* Overcoming Insomnia: Impact on Sleep, Health and Work of Online CBT-I

*Registration number:* NCT02558647

*URL*: https://clinicaltrials.gov/ct2/show/NCT02558647?cond=NCT02558647&draw=2&rank=1

**Statement of Significance**

Stabilizing sleep–wake patterns is an essential feature of several therapies, including cognitive behavioral therapy for insomnia (CBT-I). Although it is clinically thought to be essential, few have investigated whether stabilizing sleep is a feature important for therapeutic success. Our findings show that stabilizing voluntary sleep metrics do not appear to substantially mediate the effect of CBT-I itself, but are mediators in the stabilization of other sleep metrics, e.g. sleep onset latency, which in turn are important mediators of improvement in insomnia severity and especially psychological distress. These findings offer important knowledge regarding the role of stabilizing sleep in improving insomnia severity and further suggest that stabilizing sleep could be particularly beneficial for populations with high levels of psychological distress.

## Introduction

Insomnia is a major public health care concern that affects 10–12% of the general population in Western countries [1–3]. Key features of the condition include subjectively disturbed sleep alongside significant daytime distress and/or impairment [4]. Several independent randomized controlled trials (RCTs) and meta-analyses [5, 6] have demonstrated the efficacy of cognitive behavioral therapy for insomnia (CBT-I) [7] in improving sleep and psychological outcomes [5]. The strength of evidence is such that CBT-I is now the first-line treatment for insomnia [8–10]. To enhance translation of research into practice and address the clinical unmet need for interventions for insomnia, cost-efficient digital versions of CBT-I (dCBT-I) have been developed [11]. However, similar to the face-to-face CBT-I, knowledge about the benefits of dCBT-I and specific populations who most benefit from this intervention currently exceeds our understanding of how the intervention works.

It is presumed that CBT-I targets the behavioral, cognitive, and hyperarousal processes underlying insomnia and that changing these sleep regulating processes will improve the sleep disorder. In a review, nine mediators of these processes are proposed [12], including decrease time in bed, decreased napping and decrease bedtime and rise time variability. Nonetheless, knowledge is still limited on whether and which of these mediators that have a therapeutic effect. Existing reviews therefore emphasize the need to expand research in this field to understand the mechanism of action of the intervention and ensure that all CBT-I modalities include the putative therapeutic components [8, 12]. Thus, appropriate mediation analyses in RCTs to assess causality of these modalities are warranted.

We do not know which CBT-I components that are most important for therapeutic success. However, studies show that all efficacious CBT-I interventions include sleep hygiene [13], stimulus control [14], and sleep restriction [15]. These components target sleep–wake abnormalities associated with insomnia, namely bedtime (BT), rise time (RT), time in bed (TIB), sleep onset latency (SOL), wake after sleep onset (WASO), and total sleep time (TST). Given their perceived importance to clinical progress, individuals are encouraged to undertake self-monitoring of these metrics during intervention. Although these daily ratings record information about each item separately, many of these sleep metrics are inter-related (e.g. TIB = time between BT and RT). Furthermore, researchers have identified that subgroupings of these metrics can be employed to gain important insights into changes in insomnia symptoms over time and/or following exposure to different therapies [16, 17]. For instance, some sleep metrics, such as BT, RT and TIB represent voluntarily controlled behaviors (so-called “behavioral indices” (BI)), while others, such as SOL, WASO, and TST, represent involuntary controlled indices of sleep disturbance (so-called “sleep disturbance indices” (SI)).

A further issue in examining mediators is the need to select a variable or set of variables that best represent an underlying pathophysiological process that might be changed by therapy. One hypothesized behavioral mediator of CBT-I is stabilizing the sleep–wake cycle [12]. Until the last decade, the individual sleep–wake cycle over time has been mainly evaluated by intraindividual mean (IIM) values of sleep metrics. However, these IIM values represent only one element of an individual’s sleep–wake-pattern, and it is increasingly clear from basic science and clinical observational studies [18] that intraindividual variability (IIV; i.e. within-person night-to-night variation in sleep–wake patterns) can offer a more nuanced and critical insight into “healthy sleep” and sleep quality. This is easily understood when considering two individuals with a weekly average RT at 06:15 hh:mm, but one individual shows a range from 06:05 hh:mm to 06:45 hh:mm while the other shows a range from 04:45 hh:mm to 08:00 hh:mm (see Figure S1). From the perspective of the IIM, these individuals cannot be differentiated, but from the perspective of sleep variability, it is clear that they have notably different sleep–wake patterns and research findings demonstrate that higher levels of IIV are related to a wide range of negative health consequences, including insomnia severity [18]. As such, IIV is increasingly considered instead of, or at least alongside, IIM in sleep research [18].

To the best of our knowledge, only five previous CBT-I trials (one with dCBT-I) have reported IIV in sleep–wake patterns [17, 19–22].The RCTs diverged in sample sizes and sleep metrics reported, but the findings indicated that IIV was reduced pre-to-post intervention. Four of these trials examined by correlation analysis, whether reducing IIV in sleep–wake patterns after CBT-I was associated with improved outcomes after the intervention. Decreased IIV was associated with reduction in depression [17], improved cognitive performance [21] and increased self-rated sleep quality [20]. However, reducing IIV was not significantly associated with insomnia symptom remission in a large-scale RCT [22]. Importantly, none of these trials examined to what extent pre-to-post intervention changes in IIV in sleep metrics mediate the reported clinical outcomes.

The aim of the present investigation was to expand our understanding of putative mechanisms of action of dCBT-I. Using data from a large-scale RCT of adults with insomnia and modern mediation analysis techniques, we tested whether (1) the effectiveness of dCBT-I in reducing IIV and (2) whether this reduction in IIV in selected singular and composite sleep metrics mediate the effect of dCBT-I on markers of outcome that are important to clinicians and patients, namely insomnia severity and psychological distress.

## Methods

The current study utilizes data from a recently published RCT [23] and the analyses reported were specified *a priori* in the trial protocol [24]. The RCT was pre-registered on the ClinicalTrials.gov website (NCT02558647). The article on the RCT outcome [23] and trial protocol provide full details of the methodology and procedures. However, a brief overview is provided below.

### Statement of ethics

The study protocol was approved by the Regional Committee for Medical and Health Research Ethics in South East Norway (2015/134). All subjects gave written informed consent in accordance with the Revised Declaration of Helsinki.

### Overview of the RCT

Between February 2016 and July 2018, adults from the general population in Norway were recruited through general practice and media advertisements. Individuals who gave written informed consent were randomized to a parallel group RCT comparing dCBT-I with patient education about sleep (PE). Follow-up assessments were administered 9 weeks after the participant started with the intervention. No financial compensation was provided.

Inclusion criteria were: age ≥18 years and a score of ≥12 on the Insomnia Severity Index (ISI) which has a high accuracy for detecting a diagnosis of insomnia disorder in a Norwegian sample [25]. Exclusion criteria were: currently employed doing shift work; score >10 on the Epworth Sleepiness Scale; self-reported symptoms indicating sleep apnea; and/or self-report of a medical or psychiatric diagnosis where fully automated dCBT-I may be contraindicated. Eligible participants were then given access to the baseline assessments, which comprised questionnaires in addition to sleep diaries.

### Brief overview of interventions

#### *Digital cognitive behavioral therapy for insomnia*.

The digital cognitive behavioral therapy for insomnia (dCBT-I) program is a fully automated and interactive online adaption of traditional face-to-face CBT-I and included specific components (within content “cores”) such as sleep restriction, stimulus control, cognitive restructuring, sleep hygiene and relapse prevention interventions [26–28]. A Norwegian translation of Sleep Healthy Using The Internet (SHUTi) was used [29]. The program consists of 6 cores, with participants given access to the subsequent core one week after completing the previous core. Of relevance to this study is the way dCBT-I can be used to address IIV in sleep–wake patterns. As part of the sleep restriction intervention, an individual “sleep window” (based on the information in their sleep diary) is calculated and provided to users. This “sleep window” is then adjusted at the beginning of each new core based on calculations of their sleep efficiency. Participants are encouraged to keep a fixed RT so adjustments in their “sleep window” are typically achieved by shifting the BT. Also, individuals are encouraged to maintain such a stable sleep–wake pattern, for example, through statements such as this: “*…to rise at the same time is the best way to keep a stable sleep-wake pattern which is important to improve your sleep.*”

#### Online patient education about sleep

Online patient education about sleep (PE) is provided via a static website and is widely used as a control intervention in RCTs of insomnia treatments [26, 28]. The PE website describes the prevalence, causes and impact of insomnia, gives advice about when to seek input from a doctor and includes information about basic lifestyle, environmental and behavioral strategies that may improve sleep–wake patterns [28] and may therefore be similar to “treatment as usual” for people with insomnia. The user has access to all the information on the site for the duration of the trial, but there are no interactive or tailored elements. Individuals are encouraged to instigate a fixed RT throughout the week with statements such as: “*To rise at the same time every day is the best way to reset the biological clock and to get sleep back on track.*” However, no monitoring or feedback is incorporated in the programme.

### Measures

Data was retrieved from baseline and 9-week follow-up of the following self-report ratings:

#### Insomnia severity index

The ISI [30] is recommended as a standard measure for insomnia symptoms in the assessment of sleep problems and outcome in RCTs [31]. It comprises 7 items rating the severity of sleep–wake problems, impairment in daytime functioning, and concerns about sleep problems. Each item is rated on a 0 to 4 scale indicating its severity over the preceding 14 days and a higher total score indicates greater insomnia severity.

#### Hospital anxiety and depression scale

The Hospital Anxiety and Depression Scale (HADS) [32] is a 14-item rating-scale with each item rated 0–3. The scores for 7 items assessing depressive symptoms and 7 items assessing anxiety symptoms over the preceding 7 days are combined to give a measure of severity of psychological distress (range 0–42); higher scores indicate a higher level of psychological distress. The HADS is widely used in community and clinical studies.

#### The consensus sleep diary

The Consensus Sleep Diary (CSD) [33] was used daily to estimate sleep and wakefulness (from the previous night). The diary was specifically developed for, and is widely used in, insomnia research [31]. In the present study, sleep diary entries at baseline and follow-up were not necessarily required to be consecutive, but the participants had to complete a minimum of 10 sleep diary entries within 14 days. The following sleep metrics were extracted from the CSD:BT in hh:mm, SOL in minutes, WASO in minutes, and RT in hh:mm. TIB and TST (in minutes) estimates were calculated from the daily sleep diaries.

### Statistical methods

These data were analyzed using R v4.0.4 and SPSS version 26. In analyses that estimated *p*-values, the significance value was set to <0.05.

For each individual, IIV in minutes for each sleep metric at baseline and 9-week follow-up were calculated using the R package *varian* [34]. This package implements a Bayesian variability model that uses linear mixed effects to estimate IIV, and accounts for measurement errors and trends in IIV over time. To account for the uncertainty with one IIV estimate for each individual, 100 plausible values for each individual and each sleep metric were extracted and treated as multiple imputed data [35]. These values of IIV represent estimations of individuals’ variability around their own mean. The IIV for singular metrics are denoted by e.g. vBT for the IIV of BT and vRT for the IIV of RT. In addition, we derived composite scores with each of the datasets generated by *varian* by calculating *z*-scores for each IIV metric and taking the mean of these relevant *z*-scores to a Behavioral Indices Composite Score (BI-Z) (comprising vBT, vRT, and vTIB) and Sleep disturbance Indices Composite Score (SI-Z) (comprising vSOL, vWASO, and vTST).

We used linear mixed models to test if dCBT-I was effective in reducing IIV from baseline to the 9-weeks follow-up. This strategy accounts for the correlation of individual repeated measures and is less sensitive to missing data than other options. In each extracted dataset and in separate models, the effect of dCBT-I was estimated in the IIV of each sleep metric (dependent variable). Following the recommendation of Bei et al. [18], we included the IIM at baseline of each sleep metric as a covariate. Additional covariates included age and sex. The mean difference in the respective IIV metric between the dCBT-I and control group, together with 95% CIs, were calculated by pooling the model estimates using Rubin’s rules [36]. The estimated mean differences were used to calculate between-group standardized effect sizes (Cohen’s *d*) by dividing the adjusted between-group difference by the baseline pooled standard deviation of IIV. Effect sizes with a negative valence indicate a reduction in IIV between assessments. Normality of residuals were checked by visual inspection of histograms and Q–Q plots.

To test if the effect of dCBT-I on ISI and HADS can be partially explained by changes in IIV, we used the R-package *mediation* [37]. This package includes a function, mediations(), which allowed us to conduct causal mediation analysis on the multiple datasets obtained from *varian*. *Mediation* implements a series of regression analyses and estimates the average direct effects and the average causal mediation effects of a predictor (*X*) on an outcome of interest (*Y*) considering the role of one or more variables as mediators (*M*). Initially, we considered each of the IIV sleep metrics individually as potential mediators of the effect of dCBT-I on ISI. Considering the groups of interventions as the predictor and ISI as the outcome, the role of each IIV sleep metric was considered in separate analyses. Age, sex, and the baseline values of ISI and the baseline value of the relevant IIV metrics were included as covariates as these were considered potential confounders of the association between the 9-week follow-up IIV sleep metrics and ISI. This model estimates the total effect (*c*) of dCBT-I on ISI, the average direct effects (*c′*) of dCBT-I on ISI when controlling for the mediator being assessed and the average causal mediation effects denoted here as the indirect effect (*c*-*c′*). The indirect effect can be interpreted as the effect of dCBT-I compared to PE on ISI which can be attributed to the effect that dCBT-I had on the IIV sleep metric. The 95% CI of the direct and the indirect effect were obtained via bootstrapping (1000 bootstrap samples). Percentage of mediation was calculated by dividing the indirect effect by the total effect ((*c*-*c′*)/*c*). The same model was used to estimate the mediating effect of the IIV sleep metrics in the effect of dCBT-I on HADS.

The IIV metrics were found to correlate with one another suggesting that the indirect effects estimated from the mediation analyses of separate IIV in sleep metrics may probably represent overlapping effects. We therefore considered the collective role of the BI using the BI-Z and the SI using the SI-Z. This was done using the same theoretical approach as above with group of intervention as predictor, the composite score as the mediator, and ISI and then HADS as the outcome. Age, sex, baseline ISI (or HADS) and the baseline composite score were again included as covariates.

In post hoc analyses, we considered the probable sequential nature of these metrics. Specifically, we considered it likely that dCBT-I first influences the IIV in the behavioral metrics summarized in BI-Z, which in turn influenced the IIV in the sleep disturbance metrics summarized in SI-Z, and that both BI-Z and SI-Z may impact ISI and HADS. First, we assessed our assumption that changes in BI-Z influences SI-Z once again setting up a mediation analysis with group of intervention as predictor, BI-Z as the mediator and SI-Z as the outcome. Finally, the relative influence of these two possible sequential mediators was estimated using mediation Model 6 in the PROCESS package [38]. PROCESS does not handle multiple imputed data, so for this specific analysis we quantified IIV with individual standard deviation (ISD). Using this model, we could once again estimate the total effect (*c*) and direct effect (*c′*) of dCBT-I on the outcome (ISI and HADS separately), as well as three indirect pathways. These indirect pathways include: the influence of dCBT-I on the outcome via BI-Z (denoted as M1); the influence of dCBT-I on the outcome via SI-Z (M2); and the influence of dCBT-I on the outcome via first BI-Z then SI-Z (M1 then M2). Participants with missing data of the variables included in each model, were excluded from the analysis. Of 822 who completed sleep diaries at follow-up, 5 had missing values of age and 1 had missing value of HADS score at baseline. Therefore, the mediation analyses had 817 or 816 included participants.

## Results

### Demographic, clinical baseline, and sleep diaries characteristics

We extracted data for 1720 RCT participants as reported in the Intent to Treat analyses [23] (1 participant excluded due to corrupted data of the CSD). Completed CSD at baseline was 853 (Mean 11.0 days; *SD* = 0.71) for the SHUTi group and 867 (Mean 10.3 days; *SD* = 0.68) for the PE group. Completed CSD at follow-up was 420 (Mean days 11.9; *SD* = 1.51) for the SHUTi group and 402 (Mean 10.5 days; *SD* = 1.06) for the PE group. Range of CSD for both groups for the two time points were 10–14 days.

As shown in Table 1, 68% were female and the sample mean age was about 45 years. The mean ISI score was 19.4, with a sample mean BT of 23:29 hh:mm (*SD* 1:02 h), RT of 08:01 hh:mm (*SD* 1:22 h) and TST of 5:53 h (*SD* 1:14 h) at baseline assessment. At 9-week follow-up, 35.0% of the participants did not complete the ISI and 58% did not complete the HADS.

**Table 1.** Demographic, clinical, and sleep variables characteristics at baseline of participants

		PE *N* = 853	dCBT-I *N* = 867	Total *N* = 1720
Age, Mean (*SD*), y		44.8* (13.7)	44.2^†^ (13.9)	44.5 (13.8)
Sex, No (%)	Female	571 (67)	596 (69)	1167 (68)
Employment status (%)	Full-time employment	427 (50)	427 (49)	854 (50)
	Part-time employment	120 (14)	121 (14)	241 (14)
	Unemployed	46 (5)	61 (7)	107 (6)
	Retired	74 (9)	74 (9)	148 (9)
	Student	67 (8)	69 (8)	136 (8)
	Other	119 (14)	114^‡^ (13)	233 (13)
Education, Mean (*SD*), y		16.2 (2.9)	16.4 (3.0)	16.3 (2.9)
Marital status, No (%)	Cohabitant/married	535 (63)	538^‡^ (62)	1073 (62)
Living with children, No (%)	Yes	317^‡^ (37)	303^‡^ (35)	620 (36)
ISI baseline, Mean (*SD*)		19.6 (4.0)	19.2 (3.9)	19.4 (3.9)
HADS total baseline, Mean (*SD*)		13.4 (7.2)^‡^	13.2 (6.9)	13.3 (7.1)
Sleep variables, IIM (*SD*)	BT (hh:mm)	23:29 (1:02)	23:30 (1:01)	23:29 (1:02)
	RT (hh:mm)	07:59 (1:28)	08:03 (1:17)	08:01 (1:22)
	TIB (hours)	8:13 (0:59)	8:16 (1:01)	8:15 (1:00)
	SOL (hours)	0:55 (0:47)	0:56 (0:43)	0:56 (0:46)
	WASO (hours)	0:45 (0:38)	0:46 (0:40)	0:46 (0:39)
	TST (hours)	5:51 (1:14)	5:54 (1:15)	5:53 (1:14)

ISI, Insomnia Severity Index; HADS, Hospital Anxiety and Depression Scale; IIM, intraindividual mean; BT, bedtime; RT, rise time; TIB, time in bed; SOL, sleep onset latency; WASO, wake after sleep onset; TST, total sleep time.

*7 missing values; ^†^3 missing values; ^‡^1 missing value.

Independent *t*-tests were performed to compare the individuals with and without missing at 9-week follow-up in terms of baseline characteristics as age, sex, employment status, children in the household, years of education and score of ISI and HADS. Individuals with missing data on sleep diaries (analysis 1, *n* = 898) and the ISI and HADS (analysis 2, *n* = 632) had no mean differences in ISI score but were overall younger (−4.5; 95% CI −5.8 to –3.2 and −3.3 years; 95% CI −4.7 to 2.0), had less years of education (−0.5 years; 95% CI –5.8 to –3.2 and −0.6 years; 95% CI –0.9 to –0.3) and a higher HADS score (1.5 points; 95% CI 0.8 to 2.1 and 1.1 points; 95% CI 0.4 to 1.8).

### The effectiveness of dCBT-I in reducing IIV

Linear mixed modeling showed statistically significant differences between dCBT-I and PE on IIV for all sleep metrics at 9-week follow-up, with the exception of vBT (−4 minutes, *p* = 0.229). As shown in Table 2, the largest between-group differences were estimated for vTST (Cohen´s *d* = −0.42; *p* = <0.001;), vWASO (Cohen’s *d* = −0.47, *p* = <0.001) and vSOL (Cohen’s *d* = −0.54, *p* = <0.001). Overall, dCBT-I participants reported 17.6% less vTST, 36.4% less vWASO and 40.5% less vSOL compared with PE participants.

**Table 2.** Bayesian estimates of sleep metric IIV at baseline and at 9-week follow-up with dCBT-I and PE

		Baseline *N* = 1720				9-week follow-up *N* = 822				Estimated difference			
Sleep variable group	Sleep variable	dCBT-I *N* = 867		PE *N* = 853		dCBT-I *N* = 420		PE *N* = 402					
		Mean	(95% CI)	Mean	(95% CI)	Mean	(95% CI)	Mean	(95% CI)	Mean	(95% CI)	p	Cohen´s *d* between
BI	vBT (minutes)	56	54 to 58	55	53 to 57	48	45 to 51	52	49 to 55	−4	−10 to 1	0.229	− 0.14
	vRT (minutes)	69	66 to 71	70	67 to 73	59	56 to 63	69	66 to 73	−10	−16 to −4	<0.001	− 0.23
	vTIB (minutes)	71	68 to 73	69	66 to 71	61	57 to 64	68	65 to 71	−8	−14 to −2	0.006	− 0.25
SI	vSOL (minutes)	45	43 to 48	45	43 to 47	25	22 to 28	42	38 to 45	−17	−22 to −11	<0.001	− 0.54
	vWASO (minutes)	40	39 to 42	40	38 to 42	21	19 to 24	33	31 to 35	−12	−16 to −7	<0.001	− 0.47
	vTST (minutes)	94	91 to 96	92	89 to 95	70	67 to 74	85	81 to 89	−15	−22 to −8	<0.001	− 0.42

Estimated differences are the between-group effects from the linear mixed model with the IIV for the respective sleep metric as the dependent variable. Negative values favor dCBT-I. Covariates: sex, age, and baseline intraindividual mean value of the respective sleep variable. Full model output in Table S2.

BI, Behavioral indices; SI, sleep disturbance indices; vBT, IIV in bedtime; vRT, IIV in rise time; vTIB, IIV in time in bed; vSOL, IIV in sleep onset latency; vWASO, IIV in wake after sleep onset; vTST, IIV in total sleep time.

### Change in IIV in sleep metrics as mediator of change in ISI score

**Figure 1.**
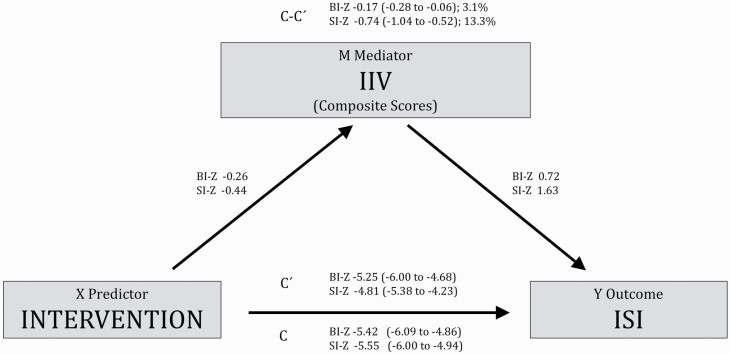
Mediation model with composite scores as mediators and Insomnia Severity Index (ISI) as outcome. Values represent unstandardized regression coefficients. Values in parentheses represent estimations of 95% confidence intervals. Values with percentage represent the estimated percentage mediated effect. Covariates: sex, age, baseline values of ISI, BI-Z, and SI-Z. BI-Z, Behavioral Indices Composite Score; SI-Z, Sleep disturbances Indices Composite Score; *c*, total effect; *c′*, average direct effects; *c*-*c′*, indirect effect. *N* = 817 (5 not included due to missing age).

As shown in Table 3, dCBT-I had a significant total effect on ISI compared with PE. Reduced vTIB, which the participant can control by behavior, was a minor, yet statistically significant, mediator accounting for an estimated 3.9% of the effect of dCBT-I on ISI. The sleep disturbance indices, vSOL, vWASO, and vTST, were also statistically significant mediators and were estimated to exert a greater mediating effect, accounting for 8.4%, 6.6%, and 5.9% of the effect of dCBT-I on ISI, respectively. However, vBT and vRT did not significantly mediate the effect of dCBT-I on ISI, each accounting for around 1% of this effect (Table 3). The composite scores also showed that the SI-Z mediated a larger proportion of the effect of dCBT-I on ISI than did the BI-Z (see Figure 1). Specifically, based on the indirect effect sizes found in this analysis, 13.3% of the effect of dCBT-I on ISI was mediated by a reduction in SI-Z (*b* = −0.74; 95% CI −1.04 to −0.52) whereas BI-Z as mediator accounted for only 3.1% (*b* = −0.17; 95% CI −0.28 to −0.06).

**Table 3.** Mediation analyses with group of intervention as predictor (*X*), ISI at 9-week follow-up as outcome (*Y*) and IIV in singular sleep metrics at 9-week follow-up as mediator (*M*)

	Mediators	Total effect (*c*)		Direct effect (*c′*)		Indirect effect (*c*-*c′*)		% Mediated
		Effect size	95% CI	Effect size	95% CI	Effect size	95% CI	
BI	vBT	−5.44	−6.14 to −4.81	−5.37	−6.07 to −4.75	−0.06	−0.16 to 0.00	1.1%
	vRT	−5.47	−5.98 to −4.86	−5.43	−5.92 to −4.74	−0.05	−0.21 to 0.06	0.9%
	vTIB	−5.42	−5.98 to −4.73	−5.21	−5.86 to −4.58	−0.21	−0.37 to −0.08	**3.9%**
SI	vSOL	−5.46	−6.15 to −4.91	−5.00	−5.71 to −4.31	−0.46	−0.64 to −0.25	**8.4%**
	vWASO	−5.44	−6.18 to −4.79	−5.09	−5.70 to −4.43	−0.36	−0.56 to −0.21	**6.6%**
	vTST	−5.45	−6.15 to −4.77	−5.13	−5.86 to −4.49	−0.32	−0.50 to −0.17	**5.9%**

Covariates: sex, age, ISI at baseline, and IIV at baseline of the specific sleep metric. *N* = 817.

BI, Behavioral indices; SI, sleep disturbance indices; vBT, IIV in bedtime; vRT, IIV in rise time; vTIB, IIV in time in bed; vSOL, IIV in sleep onset latency; vWASO, IIV in wake after sleep onset; vTST, IIV in total sleep time; CI, confidence interval. Bold text marks percentage mediation calculated from indirect effect sizes with CIs that do not cross zero.

### Change in IIV in sleep metrics as mediator of change in HADS score

**Figure 2.**
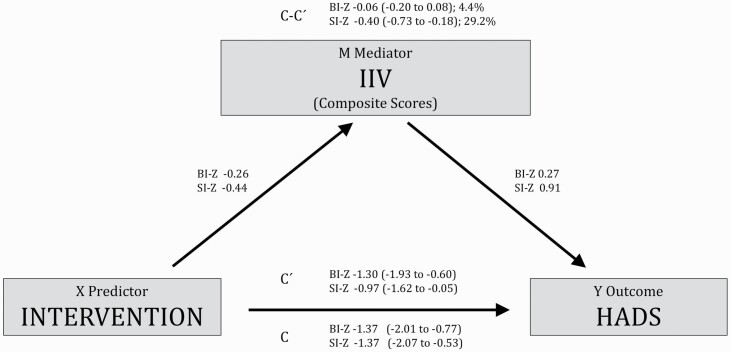
Mediation model with composite scores as mediators and Hospital Anxiety and Depression Scale (HADS) as outcome. Values represent unstandardized regression coefficients. Values in parentheses represent estimations of 95% confidence intervals. Values with percentage represent the estimated percentage mediated effect. Covariates: sex, age, baseline values of HADS, BI-Z, and SI-Z. BI-Z, Behavioral Indices Composite Score; SI-Z, Sleep disturbances Indices Composite Score; *c*, total effect; *c′*, average direct effects; *c*-*c′*, indirect effect. *N* = 816 (5 not included due to missing age and 1 due to missing HADS score at baseline).

As shown in Table 4, dCBT-I itself had a significant total effect on HADS compared with PE. Reduced vSOL, vWASO, and vTST significantly mediated the effect of dCBT-I on HADS score (accounting for 18.9%, 12.8%, and 12.6%, respectively). Reduced vRT, vBT and vTIB were estimated to only account for minor and not statistically significant mediating effects of dCBT-I on HADS (Table 4). The SI-Z mediated a substantially larger proportion of the effect of dCBT-I on HADS, compared to that mediated by BI-Z (see Figure 2). Specifically, the indirect effect sizes found in this analysis indicated that that 29.2% of the effect of dCBT-I on HADS was mediated by a reduction in SI-Z (*b* = −0.40; 95% CI −0.73 to −0.18) whereas the CI around the indirect effect meant that we could not rule out a lack of mediation by BI-Z (*b* = −0.06; 95% CI −0.20 to 0.08; 4.4%).

**Table 4.** Mediation analyses with group of intervention as predictor (*X*), HADS at 9-week follow-up as outcome (*Y*) and IIV at 9-week follow-up in different sleep variables as mediator (*M*)

	Mediators	Total effect (*c*)		Direct effect (*c′*)		Indirect effect (*c*-*c′*)		% Mediated
		Effect size	95% CI	Effect size	95% CI	Effect size	95% CI	
BI	vBT	−1.44	−2.18 to −0.93	−1.39	2.09 to −0.91	−0.05	−0.14 to 0.00	3.5%
	vRT	−1.43	−1.98 to −0.92	−1.47	−2.01 to −0.92	0.03	−0.06 to 0.14	2.1%
	vTIB	−1.32	1.96 to −0.80	−1.27	−1.83 to −0.75	−0.05	−0.14 to 0.09	3.8%
SI	vSOL	−1.43	−1.84 to −0.96	−1.16	−1.66 to −0.73	−0.27	−0.45 to −0.09	**18.9%**
	vWASO	−1.41	−2.19 to −0.76	−1.23	−2.08 to −0.56	−0.18	−0.37 to −0.02	**12.8%**
	vTST	−1.43	−2.22 to −0.77	−1.25	−1.99 to −0.59	−0.18	−0.32 to −0.04	**12.6%**

Covariates: sex, age, HADS at baseline, and IIV at baseline of the specific sleep variable. *N* = 816.

BI, Behavioral indices; SI, sleep disturbance indices; vBT, IIV in bedtime; vRT, IIV in rise time; vTIB, IIV in time in bed; vSOL, IIV in sleep onset latency; vWASO, IIV in wake after sleep onset; vTST, IIV in total sleep time; SE, standard error; CI, confidence interval. Bold text marks percentage mediation calculated from indirect effect sizes with CIs that do not cross zero.

### Sequential mediation model

When testing our assumption that changes in BI-Z influences SI-Z, the indirect effect size indicated that 20.0% of the effect of dCBT-I on SI-Z was by a reduction in BI-Z (*b =* −0.09; 95% CI −0.13 to −0.06) (see Figure S2). The sequential mediation showed a significant total effect for the intervention group on ISI (*b* = −5.45; *p* = <0.001) and HADS scores (*b* = −1.39; *p* = <0.001). Further, the indirect effect of dCBT-I on both ISI and HADS did not appear to be mediated by changes in BI-Z alone (Figure S3 and Figure S4). On the other hand, a substantial indirect effect of dCBT-I on ISI was mediated by SI-Z and via the sequential effects of dCBT-I, first on BI-Z then SI-Z (see Table S4). Similarly, BI-Z alone was found to have no statistically significant mediating effect on the impact of dCBT-I on HADS. Whereas the indirect effects mediated through SI-Z alone, and through BI-Z then SI-Z, were estimated to be small, yet statistically significant (see Table S6).

## Discussion

### Principal findings

The purpose of the present study was to test if IIV is reduced with dCBT-I, and to examine clinical implications of any identified reduction. Except from vBT, we found that dCBT-I participants experienced larger reductions in IIV on all sleep diary self-ratings compared with PE participants, especially in vSOL. To our knowledge, this is the first study to show that reduction in IIV in singular sleep metrics and composite measures significantly mediates improvements in key clinical outcomes such as insomnia severity and psychological distress. Further, reduction of sleep disturbance indices mediated greater reduction in psychological distress than insomnia severity, while change in behavioral indices was a minor and statistically non-significant mediator for these outcomes. Finally, we demonstrated a sequential pathway where reduction of behavioral indices mediated reduction in sleep disturbance indices, which in turn mediated favorable clinical outcomes.

### Interpretation

We found that dCBT-I was associated with reduced IIV in all sleep metrics, with the exception of vBT which was also observed to be reduced but did not reach statistical significance. The reduction in IIV for these sleep metrics was expected since one of the primary goals of sleep restriction is to consolidate sleep by specifically targeting and reducing IIV through the use of a weekly “sleep window” [7]. Our findings are also consistent with those of a review which demonstrated overall reduction of IIV in sleep metrics with CBT-I[18] and a recently published study using specifically dCBT-I[22]. In the present study we demonstrate that participants who received dCBT-I experienced reduced IIV across a range of individual sleep metrics compared with participants who received PE. One may speculate that because of the more personalized feedback given in an intervention of CBT-I which includes therapist support (digital or not), the IIV of the behavioral indices could be even further reduced compared with a fully automated dCBT-I. However, studies comparing IIV in these two different modalities are not known to the authors.

There are no established, clinically meaningful, thresholds of absolute values of IIV. One obvious source of heterogeneity is the sleep measures and IIV estimates employed in addition to time frame for ratings [18]. Therefore, a major limitation with existing studies have been that they only report the IIV changes and/or correlations to these [17, 19–22]. A novel contribution with the present investigation is the use of mediation analyses that can demonstrate causal effects of reducing IIV with other relevant clinical outcomes, a method not used in this field before.

Although dCBT-I itself had a greater total effect on insomnia severity relative to psychological distress, our findings showed that IIV in sleep metrics explained a greater proportion of the variance in psychological distress than insomnia severity. Few previous studies have examined the association between sleep metric IIVs and anxiety, but vTST may predict higher levels of anxiety [39, 40]. On the other hand, IIV in sleep metrics have been studied more widely in association with depression. Reducing the IIV may directly impact the circadian system and it is well established that the circadian system plays a role in the development of mood disorders [41]. Furthermore, there is evidence that IIV is associated with affective disorders [42–45] and that greater IIV in Behavioral Indices and vTST among people with insomnia may be more associated with depression than insomnia severity [17]. In a sample of adolescents, the association between mood and IIV is suggested to be mediated by perceived sleep quality [45]. However, few trials have described how a *reduction* of IIV may affect psychological distress, and the results of those that have are conflicting. Suh et al. [17] found that reduction of IIV in behavioral indices in people with chronic insomnia was associated with a reduction in depression and Manber et al. [46] found that reducing IIV in irregular sleepers led to improved mood. However, other studies did not find an association between reducing IIV and mood state [47] or mood lability [48]. Nevertheless, our findings suggest that therapeutic principles that contributes to the reduction of IIV may be particularly important to integrate in the management of psychological distress. Future studies should aim to evaluate interventions that reduce IIV on selected samples that score in the clinical range of psychological distress. Patients with acute and high degree of psychological distress are likely to be of particular interest.

Considering that dCBT-I targets the behavioral indices—especially RT—it is surprising that the reduction in IIV for these were smaller compared to the sleep disturbance indices and that they had a relatively minor role as a direct mediator to the effects of the intervention on clinical outcomes. Contrarily, reducing IIV in sleep disturbance indices, which we are unable to control by behavior, is a notable mediator of clinical outcomes. With the perspective of a person with insomnia undergoing dCBT-I, this finding may naturally weaken the adherence to the “sleep window.” Additionally, several observational studies suggest that Behavioral Indices is not important as a mediator of CBT-I [22, 45]. Interestingly, Chan et al. [16] explored mediation pathways *between* sleep metrics and found that reduced IIV in SOL and TST were mediated by reduced IIV in BT and wake time. It should be noted that the trial had a small sample size and the intervention did not include all core components of dCBT-I. However, the results from our post hoc analyses support the finding of Chan et al. as we were able to find a significant sequential mediation pathway by which Behavioral Indices mediates sleep disturbance indices that again mediates favorable changes. Our finding therefore supports a continued practice of reducing IIV in behavioral indices in dCBT-I.

In the present study, we only found partial mediation, suggesting that reducing IIV is only *one* of the mechanisms through which dCBT-I reduces insomnia severity and psychological distress. It is still largely unknown which features of dCBT-I that are most therapeutic. One study found sleep restriction therapy superior to reduction of IIV in sleep–wake patterns [49]. However, reducing IIV is to some extent, an integral part of sleep restriction therapy, making it difficult to differentiate these two components from each other. Additional comparative or mediational analyses of other core components of dCBT-I is warranted to better understand which features of the intervention that is associated with efficacy.

Overall, reducing IIV seem to be a therapeutic effect of dCBT-I that mediate favorable clinical outcomes. However, this reduction may not necessarily be important for improving clinical state on an individual level. Those with low and high IIV may be two very heterogeneous groups. For example, an individual with low IIV can have severe insomnia and high levels of rigidity concerning their sleep timing whereas another can have high IIV without experiencing any sleep disorder. Indeed, high IIV may reflect a better ability to regain sleep when needed, which for example has been shown to have beneficial effects on health compared to constantly sleeping short [50]. It should also be noted that adhering to the instructions of dCBT-I (e.g. go to bed only when feeling sleepy, see Table S1) may increase IIV in some sleep metrics for individuals who are not sleepy at their bedtime indicated by their sleep window. Whether IIV is a desired or undesired feature of sleep could be identified by the degree of predictability. One third of people with insomnia have an unpredictable sleep pattern defined as a constant median probability of having a poor night sleep [51]. Future studies that also includes this measure of the subjective experience of IIV would be of interest to distinguish the subgroup with unpredictable sleep and investigate how their reduction in IIV mediates clinical outcomes compared to those with predictable sleep.

### Strengths and limitations

The major strength of the present investigation is the use of the RCT design that reduces bias and is a robust tool to examine cause–effect relationships. Another strength is the large sample size, which gives enough statistical power to detect effects with greater precision in sub-populations. An additional asset with this study is to the use of mediation analyses, especially when clinical interpretation of absolute values of IIV is lacking. Power calculations of these analyses were not performed. Our findings should be evaluated in light of several limitations. First, participants were primarily self-referred which may introduce some self-selection biases. Second, one important potential bias were missing outcome data. At 9-week follow-up, approximately half of the participants did not complete the sleep diaries. The individuals with missing data at the 9-week follow-up had higher levels of psychological distress at baseline relative to the completers which may have affected these results. At the same time, these completion rates are typical of other CBT-I studies, and previously conducted sensitivity analyses indicate that the data are likely to be missing at random [23]. Third, mediation analyses are done without imputing missing data and can additionally be biased by hidden confounding factors between mediator and outcome. A fourth weakness with the study design is that the mediators are measured after the completion of the intervention and at the same time as the outcome variables. Future studies should include measure of sleep–wake patterns during the intervention. Fifth, a cutoff of 10 on the Epworth Sleepiness Scale was used as an exclusion criteria as it is an indicator of increased sleepiness that is associated with organic sleep disorders and individuals with insomnia have shown to have lower scores on daytime sleepiness [52]. However, this may have been too conservative [53, 54] and may have resulted in exclusion of individuals with insomnia. Sixth, the post hoc sequential mediation analyses are performed with ISD as quantification of IIV. Although comparing the Bayesian IIV method *varian* with ISD gave minor differences in our previous mediation analyses, ISD is susceptible to lower reliability [18].

## Conclusions

This RCT provides evidence that dCBT-I reduces IIV in a range of sleep metrics compared to an active control condition. Our findings demonstrate that this reduction of IIV is a therapeutic component of dCBT-I on insomnia severity and psychological distress. Although dCBT-I targets reduction of IIV in voluntary behavioral sleep metrics, these have minor *direct* mediating effect on measured clinical outcomes. However, reducing IIV in behavioral sleep metrics may still be relevant as they mediate reduced IIV in metrics of sleep disturbance which again are notable mediators of the effect of dCBT-I on both insomnia severity and psychological distress. While dCBT-I itself had a greater total effect on insomnia severity, a larger proportion of the effect of dCBT-I on psychological distress was mediated by changes in IIV. Thus, IIV may constitute a feature of sleep that is more related to psychological distress than insomnia severity itself, which further might suggest that integration of therapeutic content that contributes to reduce IIV may be important in the management of psychological distress. Determining a clinically relevant degree of reduction in IIV still needs to be established as well as the potential thresholds of IIV as clinical guidelines for professionals giving advice to patients on reducing their IIV. Future research is needed on the timeframe in which low IIV is associated with efficacy in therapy and when and for whom IIV can increase without having negative implications for clinical outcomes.

## Supplementary Material

zsab118_suppl_Supplementary_Materials_S1

zsab118_suppl_Supplementary_Materials_S2
