# Supplementary material for: The effect of sleep–wake intraindividual variability in digital cognitive behavioral therapy for insomnia: a mediation analysis of a large-scale RCT
Source: Sleep. 2021 May 8;44(10):zsab118. doi: 10.1093/sleep/zsab118 (PMC8503826; doi:10.1093/sleep/zsab118)
Supplement: zsab118_suppl_Supplementary_Materials_S1 [file zsab118_suppl_supplementary_materials_s1.pdf]

Supplemental Material

Title

The effect of sleep-wake intraindividial variability in digital cognitive behaviour therapy for insomnia: A mediation analysis of a large-scale RCT

Authors

Cecilie L. Vestergaard[1,2*], Øystein Vedaa[1,2,3,4], Melanie R. Simpson[5,6], Patrick Faaland[1,2], Daniel Vethe[1,2], Kaia Kjørstad[1,2], Knut Langsrud[2], Lee M. Ritterband[7], Børge Sivertsen[1,3,8], Tore C. Stiles[9], Jan Scott[1,10], Håvard Kallestad[1,2].

Affiliations

[1]Department of Mental Health, Norwegian University of Science and Technology, Trondheim, Norway.

[2]St. Olavs University Hospital, Østmarka, Trondheim, Norway.

[3]Department of Health Promotion, Norwegian Institute of Public Health, Bergen, Norway.

[4]Voss District Psychiatric Hospital, NKS Bjørkeli, Voss, Norway.

[5]Department of Public Health and Nursing, Norwegian University of Science and Technology, Trondheim, Norway.

[6]Clinical Research Unit Central Norway, St. Olavs Hospital, Trondheim, Norway.

[7]Center for Behavioral Health and Technology, Department of Psychiatry and Neurobehavioral Sciences, University of Virginia, Charlottesville, Virginia, USA.

[8]Department of Research & Innovation, Helse-Fonna HF, Haugesund, Norway.

[9]Department of Psychology, Norwegian University of Science and Technology, Norway.

[10]University of Newcastle, Newcastle, United Kingdom.

*Corresponding author: Cecilie Lund Vestergaard. Email: cecilie.l.vestergaard@ntnu.no

Address: AFFU, St. Olavs hospital, PO Box 3250 Torgarden, NO-7006 Trondheim, Norway

Figure S1: Intraindividual Mean (IIM) verses Intraindividual Variability (IIV).

Two participants with insomnia from our study with the exact same IIM of rise time (06:15:00) in their 10 days of sleep diary, but different degree of IIV: Participant 1 = 0:12:28 h Individual Standard Deviation (ISD) (range 06:05 to 06:45 hh:mm) and Participant 2 = 1.32.17 h ISD (range 04:45 to 08:00 hh:mm).

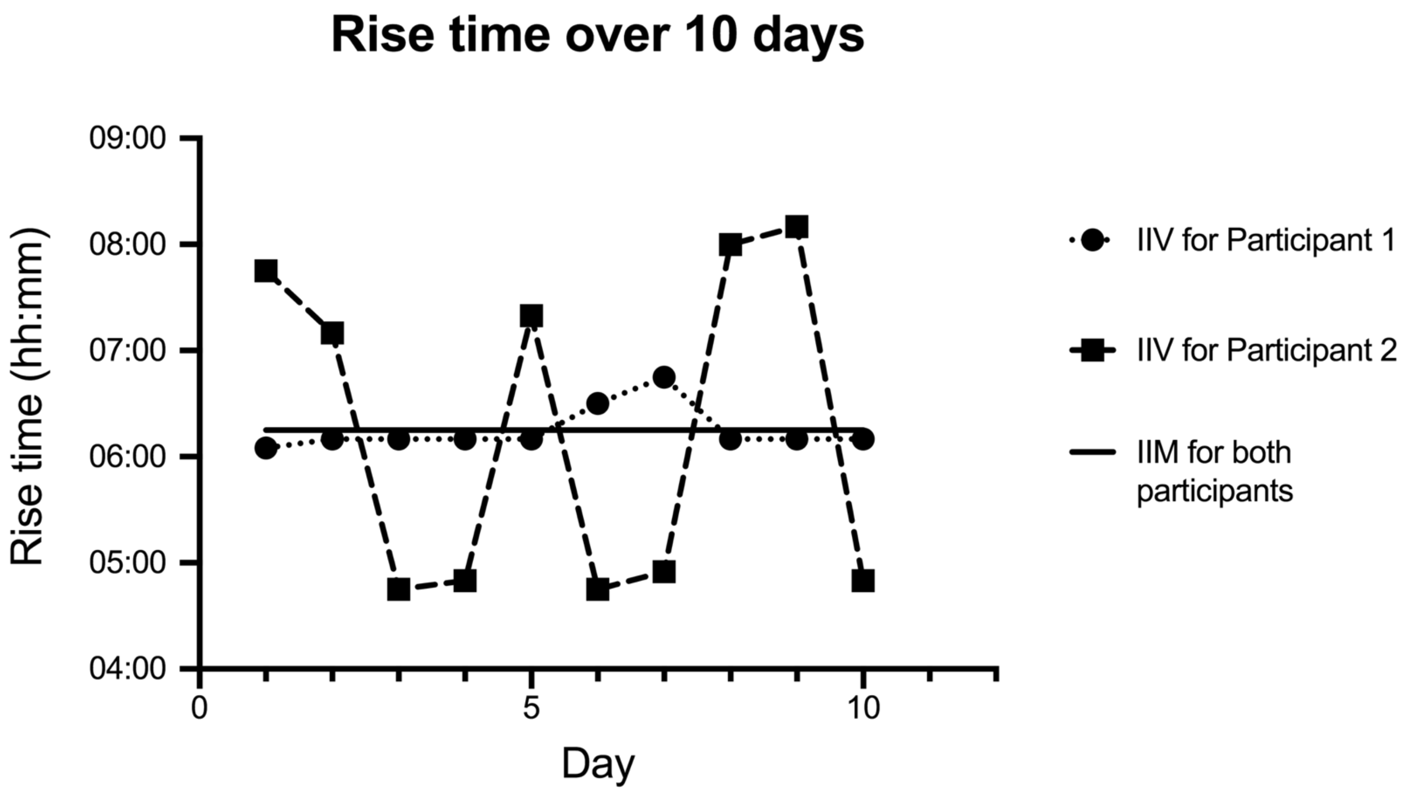

Figure S2: Mediation model with intervention group as predictor, Behavioural Indices Composite Score (BI-Z) as mediator and Sleep disturbance Indices Composite Score (SI-Z) as outcome.

Values represent unstandardized regression coefficients. Values in parentheses represent estimations of 95% confidence intervals. Values with percentage represent the estimated percentage mediated effect. Covariates: age, sex, BI-Z baseline and SI-Z baseline. N=817. c: total effect; c´: average direct effects; c-c´: indirect effect.

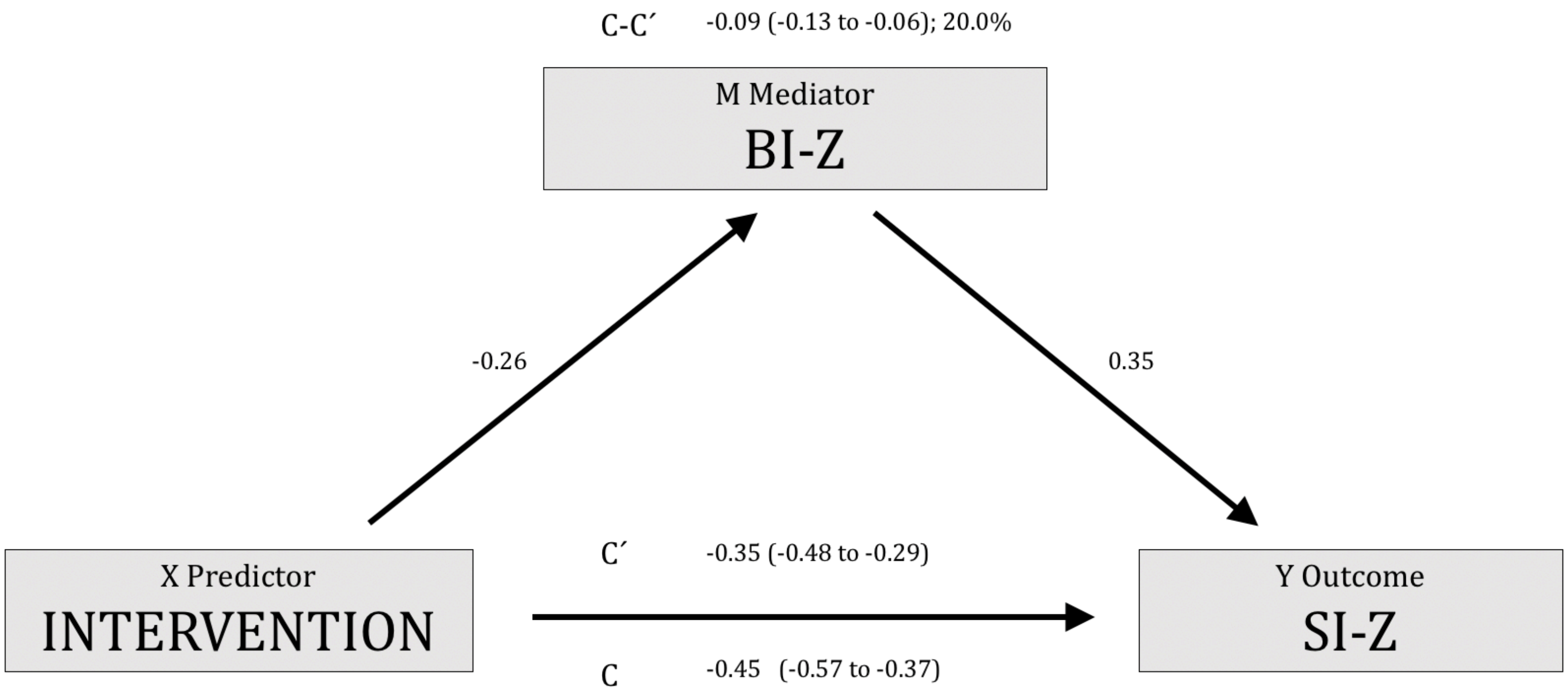

Figure S3: Sequential mediation model with Insomnia Severity Index (ISI) as outcome.

Two mediators (BI-Z as MI and SI-Z as M2) operating in serial with Insomnia Severity Index (ISI) as outcome Y and predictor X as intervention group. Covariates include sex, age, IIV at baseline of the composite scores and the baseline score of ISI. For this specific analysis, individual standard deviation (ISD) in hours was used as quantification of IIV. Values represent unstandardized regression coefficients and orange arrows represent statistically significant beta values. Values in parentheses represent total effects. N=817. BI-Z: Behavioural Indices Composite Score; SI-Z: Sleep disturbances Indices Composite Score. *: p <0.001

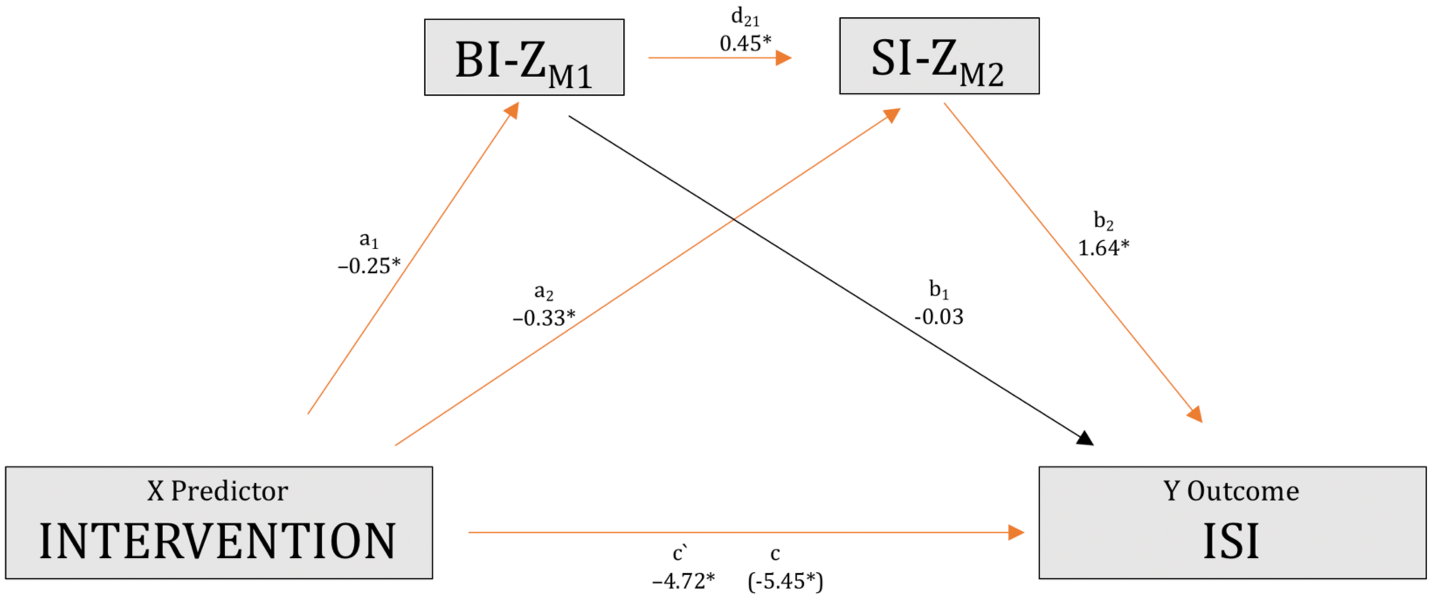

Figure S4: Sequential mediation model with Hospital Anxiety and Depression Scale (HADS) as outcome.

Two mediators (BI-Z as MI and SI-Z as M2) operating in serial with Hospital Anxiety and Depression Score (HADS) as outcome Y and predictor X as intervention group. Covariates include sex, age, IIV at baseline of the composite scores and the baseline score of HADS. For this specific analysis, individual standard deviation (ISD) in hours was used as quantification of IIV. Values represent unstandardized regression coefficients and orange arrows represent statistical significant beta values. Values in parentheses represent total effects. N=816. BI-Z: Behavioural Indices Composite Score; SI-Z: Sleep disturbances Indices Composite Score. *: p <0.001

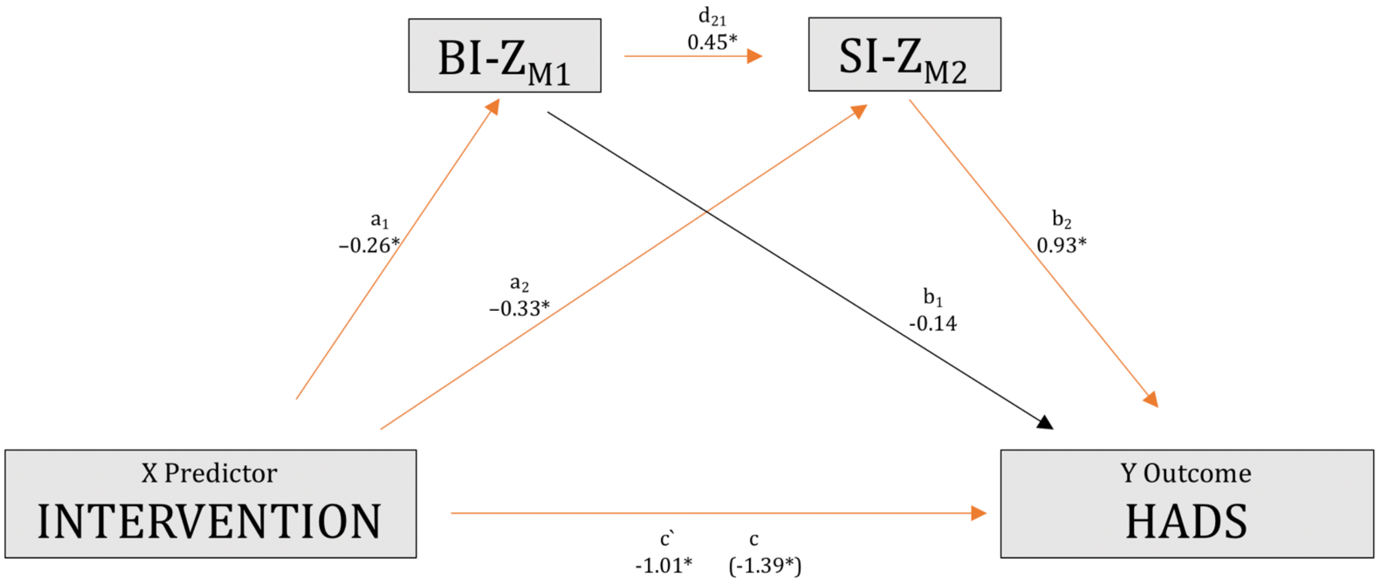

Table S1: Specific instructions in the interventions that may affect IIV.

Text in cursive is directly citations of the intervention web platforms.

Intervention	Instruction	Context of the instruction
Sleep Healthy Using the Internet (SHUTi)	Consolidated Sleep<br>Now see what a night of consolidated sleep looks like. This is a night with Sleep Restriction. Go!<br>• Delay bedtime until you feel sleepy. 10:00 – 11:00 PM Not in bed awake<br>• Go to bed feeling sleepy. Fall asleep within a half hour. 11:00 PM – 12:00 AM Not in bed awake | In bed awake | In bed asleep<br>• Stay asleep. 12:00 – 5:00 AM<br>• Wake when the alarm goes off. 5:00 AM<br>• Get up and out of bed for the day. 5:00 AM<br>Follow your Sleep Window carefully!	*Core 2*<br>*Info about sleep scheduling*
	You will arise at this time every day including weekends. It is extremely important to get out of bed (arise) as soon as you wake up in the morning. It is very important that you get out of bed at your Arising Time, regardless of how much sleep you had. This is true even if you fell asleep only three hours before your scheduled Arising Time. This is a *very* important part of this program. Sticking to this part of the program greatly increases your chances of success.	*Selecting "arise time"*
	Your Sleep Window is the maximum number of hours you are to stay in bed each night. After you select your Arising Time, your Bedtime is automatically calculated by counting back the number of hours in your Sleep Window. This Bedtime will be the earliest time you are to go to bed.	*Info about bedtime*

	RULE 3: STAY AWAKE UNTIL YOUR SLEEP WINDOW BEDTIME<br>What if you can't stay awake until your bedtime? This is a good sign! For example, if your Sleep Window Bedtime is midnight, you should not go to bed before that time even though you feel as if you could fall asleep at 11:00 pm. You may have to fight your sleepiness in order to stick to your Sleep Window.<br><br>RULE 4: GO TO BED ONLY WHEN SLEEPY<br>You might find yourself struggling to stay awake until your bedtime, but when your Sleep Window finally arrives, you are wide awake. When this happens, it is important to stay up until you start to feel sleepy. Your Sleep Window is a period of time you *may* sleep, not *must* sleep. Feeling wide awake at your Sleep Window Bedtime is usually a temporary problem. Be sure to go to bed only when you are sleepy (and during your Sleep Window).<br><br>RULE 5: GET OUT OF BED IF YOU CAN'T SLEEP<br>Never stay in bed awake for more than 15-20 minutes. If you feel sleepy but then get in bed and can't fall asleep within 15-20 minutes, get out of bed. When you wake in the middle of the night and can't fall asleep within that time, get out of bed. You are trying to teach your body and mind that bed is a place for sleep. Staying in bed when you are awake gives your body and mind the wrong message.<br><br>RULE 6: KEEP A REGULAR ARISING TIME<br>Keep your Arising Time the same. Set your alarm clock for your Arising Time and get out of bed every morning, weekdays and weekends, no matter how much you slept the previous night. Although it may be tempting to stay in bed later because you did not sleep well, or to stay in bed later on weekends, try to maintain a steady sleep schedule. This is the	*Core 2 and Core 3*<br><br>*At the end of core 2, the "Ground Rules" are explained like this: "Ground Rules for sleep are a set of behavioral practices and strategies that are necessary to follow for success in improving sleep."*<br><br>*Participants are instructed to follow these in Core 2 and 3. Additionally, these rules are reinforced through video case vignettes.*

	best way to reset your biological clock and get your sleep back on track.<br><br>RULE 7: NO NAPPING<br>Although many people with sleep problems try to nap to make up for lost sleep, this strategy often backfires. For people with insomnia, napping can disrupt the sleep-wake rhythm and interferes with nighttime sleep. You will learn more about this in Core 3 (Sleep Practices). Remember that staying awake during the day will help you feel sleepier at night and increase your chance of falling asleep.	
Patient Education (PE)	1. Go to bed only when sleepy<br>People with insomnia often go to bed too early in an attempt to make sure they fall asleep by the desired time. Going to bed too early, especially if you are not sleepy, can make things worse. The bed and bedroom surroundings become cues for wakefulness rather than for sleepiness. Also, going to bed too early allows more time to worry about difficulty falling asleep.<br><br>2. If you don't fall asleep after 20 minutes, get out of bed<br>The guideline to get out of bed if you don't fall asleep after 20 minutes should be followed whether sleeplessness occurs when going to sleep, in the middle of the night, or in the morning. Getting out of bed and leaving the bedroom to engage in quiet activity such as reading, listening to music, watching a movie or any other non-stimulating activities may be helpful at such times. It is also recommended to wait until feeling sleepy before going back to bed.<br><br>3. Maintain a regular arising time in the morning<br>The best way to reset the biological clock and get sleep back on track is to maintain a regular arising time in the morning. However, it can be difficult to keep a regular arising time every morning, especially on the weekend. Although it may be tempting to stay in bed later because of bad sleep quality, or	*"Insomnia and sleep habits" guidelines 1, 2, 3, 4 out of 5. Below the guidelines it is stated:*<br>*"Together, the above recommendations help the body and mind associate the bed as a place for sleep."*

	to stay in bed later on weekends, a steady sleep schedule can help improve sleep.<br><br>4. Limit daytime napping<br>For people with insomnia, napping disrupts the sleep-wake rhythm and interferes with nighttime sleep. Although staying awake all day can be hard, the aim of avoiding naps during the day is to feel sleepier at night and thereby increase the chance of falling asleep at night.	

Table S2: Intercept of mixed models with Bayesian estimates of IIV as the dependent variable as summarized in Table 2.

Time; 0=Baseline, 1=Follow-up. Treatment; 0=dCBT-, 1= PE. Age variable not centered.

vBT = IIV in Bedtime; vRT = IIV in Rise Time; vTIB = IIV in Time In Bed; vSOL= IIV in Sleep Onset Latency; vWASO = IIV in Wake After Sleep Onset; vTST = IIV in Total Sleep Time; IIM = Intraindividual mean

Dependent variable	Term	Estimate	SE	Statistic	df	p.value
vBT	Intercept	-1.841389224	0.2768695467	-6.6507467	1224.395	<0.001
vBT	time	-0.126631874	0.0289612859	-4.3724534	1183.591	<0.001
vBT	treatment	-0.008539530	0.0261533238	-0.3265180	1162.673	0.744
vBT	age	-0.008510191	0.0008401182	-10.1297533	1213.280	<0.001
vBT	sex	0.015190718	0.0239070721	0.6354069	1692.532	0.525
vBT	IIM of BT	0.134169947	0.0115712505	11.5951122	1154.950	<0.001
vBT	time:treatment	0.077354854	0.0412959803	1.8731812	1181.446	0.061
vRT	Intercept	0.64064036	0.10298731	6.220576	945.8702	<0.001
vRT	time	-0.15782797	0.03067745	-5.144755	1203.8343	<0.001
vRT	treatment	0.01787385	0.02946436	0.606626	947.3183	0.544
vRT	age	-0.01021605	0.00095498	-10.697654	1241.1776	<0.001
vRT	sex	0.03653609	0.02705589	1.350393	1387.2375	0.177
vRT	IIM of RT	0.11960299	0.01033828	11.568944	762.4856	<0.001
vRT	time:treatment	0.14701272	0.04585293	3.206180	828.7015	0.001
vTIB	Intercept	0.812431783	0.1219094242	6.6642246	1016.1018	<0.001
vTIB	time	-0.171677413	0.0307934952	-5.5751194	1094.0516	<0.001
vTIB	treatment	-0.030834326	0.0277504142	-1.1111303	1203.8908	0.266
vTIB	age	-0.008611856	0.0008914265	-9.6607581	1377.2466	<0.001
vTIB	sex	0.023432187	0.0265474976	0.8826514	1182.0843	0.378
vTIB	IIM of TIB	0.090231565	0.0130541675	6.9120888	869.0118	<0.001

vTIB	time:treatment	0.159790094	0.0440253086	3.6295054	1071.3355	<0.001
vSOL	Intercept	0.122228427	0.041924423	2.915447	987.4941	0.004
vSOL	time	-0.345337368	0.029539660	-11.690635	1600.5995	<0.001
vSOL	treatment	-0.006565088	0.024698141	-0.265813	1230.5058	0.790
vSOL	age	0.001701901	0.000754979	2.254236	1053.4286	0.024
vSOL	sex	-0.027827517	0.021718076	-1.281307	1335.4011	0.020
vSOL	IIM of SOL	0.621975662	0.017740121	35.060396	302.5578	<0.001
vSOL	time:treatment	0.284952959	0.042267771	6.741613	1537.2946	<0.001
vWASO	Intercept	0.314886983	0.0307463662	10.2414374	1476.3876	<0.001
vWASO	time	-0.319811796	0.0242254769	-13.2014655	1503.4466	<0.001
vWASO	treatment	-0.009645234	0.0204917814	-0.4706879	1154.8488	0.637
vWASO	age	0.000434055	0.0006311391	0.6877328	1331.9960	0.491
vWASO	sex	-0.030201230	0.0178856213	-1.6885760	1474.3401	0.091
vWASO	IIM of WASO	0.457734956	0.0153602500	29.7999679	518.5313	<0.001
vWASO	time:treatment	0.207192947	0.0345141815	6.0031250	1501.2586	<0.001
vTST	Intercept	2.010559695	0.0957620535	20.9953695	762.9476	<0.001
vTST	time	-0.390311315	0.0350410050	-11.1387021	906.1261	<0.001
vTST	treatment	-0.024897727	0.0306751273	-0.8116585	972.2651	0.417
vTST	age	-0.004728519	0.0009938294	-4.7578783	1115.9142	<0.001
vTST	sex	-0.048027634	0.0288926631	-1.6622778	950.4152	0.096
vTST	IIM of TST	-0.036919077	0.0118208210	-3.1232244	723.0578	0.002
vTST	time:treatment	0.276986504	0.0512075945	5.4090903	757.7067	<0.001

Table S3: Model output from the sequential mediation model with ISI as outcome illustrated in Figure S3.

Path	b	(df2) = t	SE	p-value	R^2	95% CI
a_1	-0.25	(810) = -4.87	0.05	p<0.001	0.25	-0.35 to -0.15
a_2	-0.33	(809)= -7.60	0.04	p<0.001	0.38	-0.42 to -0.25
b_1	-0.03	(808)= -0.11	0.26	0.91	0.37	-0.55 to 0.49
b_2	1.64	(808)=5.87	0.28	p<0.001	0.37	1.09 to 2.19
d_{21}	0.45	(809) = 15.30	0.03	p<0.001	0.38	0.39 to 0.51
c (Total effect)	-5.45	(810)=-15.47	0.35	p<0.001	0.33	-6.14 to 4.76
c´ (Direct effect)	-4.72	(808)=-13.09	0.36	p<0.001	0.37	-5.43 to -4.01

Table S4: Unstandardized indirect effect sizes of the different pathways in the sequential mediation model illustrated in Figure S3.

Text in cursive indicates indirect effect sizes with CI´s that do not cross zero.

Pathway	*b*	SE	95% CI
X > BI-Z > Y	0.01	0.07	-0.13 to 0.15
X > SI-Z > Y	*-0.55*	0.11	-0.79 to -0.34
X > BI-Z > SI-Z > Y	*-0.19*	0.05	-0.30 to -0.09
Total indirect effects of X on Y	*-0.72*	0.13	-0.98 to -0.49

Table S5: Model output from the sequential mediation model with HADS as outcome illustrated in Figure S4.

Path	b	(df2) = t	SE	p-value	R^2	95% CI
a_1	-0.26	(809)=-4.93	0.05	p<0.001	0.25	-0.36 to -0.15
a_2	-0.33	(808)=-7.53	0.04	p<0.001	0.38	-0.42 to -0.24
b_1	-0.14	(807)=-0.57	0.25	0.57	0.55	-0.63 to 0.35
b_2	0.93	(807)=3.52	0.26	p<0.001	0.55	0.41 to 1.45
d_{21}	0.45	(808)=15.42	0.03	p<0.001	0.38	0.39 to 0.51
c (Total effect)	-1.39	(809)=-4.25	0.33	p<0.001	0.54	-2.03 to -0.75
c´ (Direct effect)	-1.01	(807)=-2.97	0.34	p<0.001	0.55	-1.68 to -0.34

Table S6: Unstandardized indirect effect sizes of the different pathways in the sequential mediation model illustrated in Figure S4.

Text in cursive indicates indirect effect sizes with CI´s that do not cross zero.

Pathway	*b*	SE	95% CI
X > BI-Z > Y	0.04	0.07	-10 to 0.19
X > SI-Z > Y	*-0.31*	0.10	-0.51 to -0.11
X > BI-Z > SI-Z > Y	*-0.11*	0.04	-0.20 to -0.04
Total indirect effects of X on Y	*-0.38*	0.11	-0.60 to -0.15

Table S7: Mediator model (model m) and outcome model (model y) estimated by *mediations()* in mediation analyses presented in Figure 1, Figure 2, Table 3, Table 4 and Figure S2. The mediator and outcome for each analysis is specified, predictor in all analyses are intervention group.

**Mediator**	**Outcome**	**Model**	**Coefficients**						
vBT post	ISI	model y	Intercept	Intervention	vBT follow-up	ISI baseline	Sex	Age	vBT baseline
n=817			1.786725	-5.372361	0.932686	0.573558	0.979520	0.008253	-0.278416
		model m	Intercept	Intervention	ISI baseline	Sex	Age	vBT baseline	
			0.749431	-0.064099	0.003192	0.030741	-0.004777	0.225494	
vBT post	HADS	model y	Intercept	Intervention	vBT follow-up	HADS baseline	Sex	Age	vBT baseline
N=816			3.192776	-1.361412	0.708039	0.692815	-0.002229	-0.026282	-0.343750
		model m	Intercept	Intervention	HADS baseline	Sex	Age	vBT baseline	
			0.766947	-0.065116	0.002206	0.029211	-0.004383	0.225691	
vRT post	ISI	model y	Intercept	Intervention	vRT follow-up	ISI baseline	Sex	Age	vRT baseline
N=817			2.297783	-5.391430	0.240406	0.575608	0.995162	0.005153	-0.153271
		model m	Intercept	Intervention	ISI baseline	Sex	Age	vRT baseline	
			0.866689	-0.169347	0.003205	0.048743	-0.006050	0.366941	
vRT post	HADS	model y	Intercept	Intervention	vRT follow-up	HADS baseline	Sex	Age	vRT baseline
N=816			4.03146	-1.43621	-0.17529	0.69562	0.02560	-0.03132	-0.18124
		model m	Intercept	Intervention	HADS baseline	Sex	Age	vRT baseline	
			0.901919	-0.170384	0.001406	0.047885	-0.005782	0.364989	
vTIB post	ISI	model y	Intercept	Intervention	vTIB follow-up	ISI baseline	Sex	Age	vTIB baseline
N=817			1.786221	-5.210403	1.351976	0.571268	1.047210	0.006449	-0.857273
		model m	Intercept	Intervention	ISI baseline	Sex	Age	vTIB baseline	

			1.004938	-0.157508	0.004279	-0.025050	-0.004686	0.253758	
vTIB post	HADS	model y	Intercept	Intervention	vBT follow-up	HADS baseline	Sex	Age	vBT baseline
N=816			3.48461	-1.33613	0.43675	0.69391	0.02601	-0.02830	-0.41179
		model m	Intercept	Intervention	HADS baseline	Sex	Age	vBT baseline	
			1.005512	-0.159317	0.004513	-0.027168	-0.003980	0.249905	
vSOL post	ISI	model y	Intercept	Intervention	vSOL follow-up	ISI baseline	Sex	Age	vSOL baseline
N=817			1.54237	-4.98886	1.64373	0.57126	1.09763	0.00655	-0.50262
		model m	Intercept	Intervention	ISI baseline	Sex	Age	vSOL baseline	
			0.521945	-0.270637	0.003170	-0.056200	-0.001298	0.307410	
vSOL post	HADS	model y	Intercept	Intervention	vSOL follow-up	HADS baseline	Sex	Age	vSOL baseline
N=816			3.16909	-1.15620	0.92498	0.69081	0.05246	-0.02683	-0.45544
		model m	Intercept	Intervention	HADS baseline	Sex	Age	vSOL baseline	
			0.492811	-0.268981	0.005269	-0.064016	-0.000554	0.303325	
vWASO post	ISI	model y	Intercept	Intervention	vWASO follow-up	ISI baseline	Sex	Age	vWASO baseline
N=817			1.910e+00	-5.089e+00	2.183e+00	5.677e-01	1.071e+00	7.122e-05	-5.894e-01
		model m	Intercept	Intervention	ISI baseline	Sex	Age	vWASO baseline	
			0.225227	-0.157695	0.004143	-0.030474	0.002019	0.265426	
vWASO post	HADS	model y	Intercept	Intervention	vWASO follow-up	HADS baseline	Sex	Age	vWASO baseline
N=816			3.35481	-1.21160	1.18019	0.69369	0.01681	-0.02741	-0.74066
		model m	Intercept	Intervention	HADS baseline	Sex	Age	vWASO baseline	
			3.001e-01	-1.581e-01	-4.546e-05	-3.195e-02	2.095e-03	2.707e-01	

vTST post	ISI	model y	Intercept	Intervention	vTST follow-up	ISI baseline	Sex	Age	vTST baseline
N=817			0.812681	-5.081988	1.357261	0.570744	1.017569	0.009026	-0.296673
		model m	Intercept	Intervention	ISI baseline	Sex	Age	vTST baseline	
			1.234316	-0.260298	0.004967	-0.009700	-0.003490	0.171587	
vTST post	HADS	model y	Intercept	Intervention	vTST follow-up	HADS baseline	Sex	Age	vTST baseline
N=816			3.9130362	-1.2538259	0.6807975	0.6955244	0.0003386	-0.0266058	-0.9276083
		model m	Intercept	Intervention	HADS baseline	Sex	Age	vTST baseline	
			1.252682	-0.261227	0.003932	-0.013353	-0.002825	0.172548	
SI-Z post	ISI	model y	Intercept	Intervention	SI-Z post	ISI baseline	Sex	Age	SI-Z baseline
N=817			2.078080	-4.718879	1.633905	0.564439	1.109605	0.005598	-0.529973
		model m	Intercept	Intervention	ISI baseline	Sex	Age	SI-Z baseline	
			0.190813	-0.436922	0.008029	-0.065285	-0.000769	0.309469	
SI-Z post	HADS	model y	Intercept	Intervention	SI-Z post	HADS baseline	Sex	Age	SI-Z baseline
N=816			3.18748	-1.00046	0.91256	0.69290	0.02144	-0.02560	-0.76505
		model m	Intercept	Intervention	HADS baseline	Sex	Age	SI-Z baseline	
			2.518e-01	-4.372e-01	4.942e-03	-7.211e-02	7.599e-05	3.134e-01	
BI-Z post	ISI	model y	Intercept	Intervention	BI-Z post	ISI baseline	Sex	Age	BI-Z baseline
N=817			2.299359	-5.240344	0.718222	0.571223	0.994855	0.006993	-0.454566
		model m	Intercept	Intervention	ISI baseline	Sex	Age	BI-Z baseline	
			0.360912	-0.257988	0.007162	0.029671	-0.008650	0.375026	
BI-Z post	HADS	model y	Intercept	Intervention	BI-Z post	HADS baseline	Sex	Age	BI-Z baseline

N=816			3.52618	-1.33124	0.27447	0.69448	0.01329	-0.02941	-0.30809
		model m	Intercept	Intervention	HADS baseline	Sex	Age	BI-Z baseline	
			0.405293	-0.260774	0.004755	0.027506	-0.007827	0.372867	
BI-Z post	SI-Z post	model y	Intercept	Intervention	BI-Z post	Sex	Age	SI-Z baseline	BI-Z baseline
N=817			0.155517	-0.346320	0.353670	-0.080122	0.002699	0.292084	-0.123302
		model m	Intercept	Intervention	Sex	Age	SI-Z baseline	BI-Z baseline	
			0.514764	-0.259474	0.035880	-0.009157	0.072108	0.352670	

ISI: Insomnia Severity Index; HADS: Hospital Anxiety and Depression Scale; vBT = IIV in Bedtime; vRT = IIV in Rise Time; vTIB = IIV in Time In Bed; vSOL= IIV in Sleep Onset Latency; vWASO = IIV in Wake After Sleep Onset; vTST = IIV in Total Sleep Time; BI-Z: Behavioural Indices Composite Score; SI-Z: Sleep disturbances Indices Composite Score.
