# Supplementary material for: The effect of sleep–wake intraindividual variability in digital cognitive behavioral therapy for insomnia: a mediation analysis of a large-scale RCT
Source: Sleep. 2021 May 8;44(10):zsab118. doi: 10.1093/sleep/zsab118 (PMC8503826; doi:10.1093/sleep/zsab118)
Supplement: zsab118_suppl_Supplementary_Materials_S2 [file zsab118_suppl_supplementary_materials_s2.pdf]

```
##################################################################################
##  R SCRIPT: RISE TIME: AN EXAMPLE FROM SLEEP DIARY DATA TO IIV        ##
##  ESTIMATES WITH VARIAN AND ANALYSES WITH                                  ##
##  LMER AND MEDIATIONS                                                      ##
##################################################################################


### CREDIT: Made with an example kindly provided by Joshua F. Wiley. ###


####### IVV ESTIMATION WITH VARIAN #######

### BASELINE ESTIMATION

# Importing data file in long format with all sleep diaries at baseline
baseline <- read.csv("r_file_baseline.csv")

## varian wants a data frame
baseline <- as.data.frame(baseline)
## order by ID
baseline <- baseline[order(baseline$id)]


## this is just some sample "wide" data for now
widedata_arise_baseline <- subset(baseline, !duplicated(id))[, c("id", "participant_type", "age_n",
  "sex_numerical", "pre_ariseMean_n")]

library(varian)

## main bayesian IIV estimates
options(mc.cores = 4L)
m_arise_baseline <- varian(v.formula = arise_n ~ 1 | id,
                     data = as.data.frame(baseline), design = "V",
                     useU = TRUE, totaliter = 6000, warmup = 1000, thin = 2,
                     chains = 4)

## these give some visual diagnostic plots
vm_diagnostics(m_arise_baseline)

## convert posterior estimates into a data frame
## different parameters for different IDs are columns
res_arise_baseline <- as.data.frame(m_arise_baseline$results)

## get just the variability estimates
iivs_arise_baseline <- grep("Sigma_V.*", names(res_arise_baseline), value = TRUE)
iivdata_arise_baseline <- res_arise_baseline[, iivs_arise_baseline]

## there should be one variability estimate per ID so this should be TRUE
## just a quality check
ncol(iivdata_arise_baseline) == length(unique(baseline$id))


## We chose to sample 100
set.seed(1234) ## make random sample reproducible
```

```
useposteriors_arise_baseline <- sample(1:nrow(res), size = 100, replace = FALSE)

## making one datalist with 100 data frames with our selected wide variables
## (the same in each dataframe for each ID) and
## the IIV estimates (different in each dataframe)
listres_arise_b <- lapply(useposteriors_arise_baseline, function(i) {
  tmpdata <- data.frame(
    Imputation = i,
    id = unique(baseline$id),
    IIV_arise_b = as.numeric(unlist(iivdata_arise_baseline[i, ])))
  merge(tmpdata, widedata_arise_baseline, by = "id", all = TRUE)
})


#### Manually including those with 0 in IIV. (Varian would not run without
#### excluding these, even though we tried with opts = list(SD_Tol = 0, pars=NULL))

test <- widedata_arise_baseline

## Making this file to a list with 100 of the same list
test2 = replicate(n = 100,
                  expr = {data.frame(test)},
                  simplify = F)

## then merge inn the IIV data from listres_arise_p in the testfile

test3 <- test2
for (i in 1:100)  {
  test3[[i]]<- merge(test3[[i]], listres_arise_b[[i]], by=c("id", "participant_type", "age_n",
 "sex_numerical", "pre_ariseMean_n"), all=TRUE )
  test3[[i]]$IIV_arise_b[is.na(test3[[i]]$IIV_arise_b)] <- 0
  test3[[i]]$Imputation[is.na(test3[[i]]$Imputation)] <- 0
}

listres_arise_b <- test3

listres_arise_b <- lapply(listres_arise_b, "[", -grep(c("Imputation"), names(listres_arise_b[[1]])))



## first few rows of imputed dataset 1
head(listres_arise_b[[1]])

## first few rows of imputed dataset 5; note values slightly different
## than #1, that's because of uncertainty in estimating an individual person's IIV
head(listres_arise_b[[5]])


library(mice) ## package for analyzing multiply imputed data
library(miceadds) ## package with some utilities for manipulating imputed datasets

impres_arise_b <- miceadds::datlist2mids(listres_arise_b)

## this should be true
is.mids(impres_arise_b)
```

FOLLOW-UP ESTIMATIONS OF IIV

Doing the same steps as for baseline estimations

```
####### MIXED MODELS #######


## In listres_arise_b
## Rename column IIVariseb to IIVarise in all dataframes
## Create new column called time = 0 for all dataframes

mixlist_ariseb <- listres_arise_b
for (i in 1:100)  {
  names(mixlist_ariseb[[i]])[names(mixlist_ariseb[[i]]) == "IIV_arise_b"] <- "IIV_arise"
  mixlist_ariseb[[i]]$time <- 0
}

## In listres_arise_p
## Rename column IIVarisep to IIVarise in all dataframes
## Create new column called time = 1 for all dataframes

mixlist_arisep <- listres_arise_p
for (i in 1:100)  {
  names(mixlist_arisep[[i]])[names(mixlist_arisep[[i]]) == "IIV_arise_p"] <- "IIV_arise"
  mixlist_arisep[[i]]$time <- 1
}

## Append pre and post datasets for each 100
mixlist_arise <-  mixlist_arisep
for (i in 1:100)  {
  mixlist_arise[[i]] <-  rbind(mixlist_arisep[[i]], mixlist_ariseb[[i]])
}

 ftable(mixlist_arise[[1]]$time)


 for(i in 1:length(mixlist_arise))   {
   mixlist_arise[[i]] <- mixlist_arise[[i]] %>%
     mutate(treatment = recode(participant_type,
                          "Control" = 1,
                          "Experimental" = 0)) }



 library(mice) ## package for analyzing multiply imputed data
 library(miceadds) ## package with some utilities for manipulating imputed datasets

 mixedimpres_arise <- miceadds::datlist2mids(mixlist_arise)

## this should be true
 is.mids(mixedimpres_arise)

 ftable(mixedimpres_arise[[1]]$time)

## does sleep diary arise IIV differ by Condition?
model.results_arise <- with(mixedimpres_arise, lmer(IIV_arise ~ time*treatment + age_n +
  factor(sex_numerical) + pre_ariseMean_n + (1|id)))

## pool and summarize the results
```

```
summary(pool(model.results_arise))


## make emmeans object
emm_model.results_arise <- emmeans(model.results_arise, ~ time:treatment)
summary(emm_model.results_arise)

## summary of pairs with CI
summary(pairs(emm_model.results_arise), infer = c(TRUE, TRUE))
```

```
######## MEDIATION ########


library(mediation)
library (acepack)
library (latticeExtra)
library (Hmisc)

# Importing data file and save it as a data.frame
wide <- read.csv("r_mediation_wide5.csv")



##### ISI

## merging lists from the IIV estimation in varian (baseline and follow up) to
## one datalist mergelistres_arise

mergelistres_arise <- listres_arise_p
for (i in 1:100)  {
  mergelistres_arise[[i]] <- merge(mergelistres_arise[[i]], wide, all = TRUE, by=c("id",
 "participant_type"))
  names(mergelistres_arise[[i]])[names(mergelistres_arise[[i]]) == "treatment"] <- "r"
}

for (i in 1:100)  {
  mergelistres_arise[[i]] <- merge(mergelistres_arise[[i]], listres_arise_b[[i]], all = TRUE, by=c("id",
 "participant_type"))
}


## have to give the datalist in mergelistres_arise a name for each list
## and define this for usage in mediations
## each data set has its respective treatment variable.

datasets <- list(
  IMP1=mergelistres_arise[[1]],
  IMP2=mergelistres_arise[[2]],
  IMP3=mergelistres_arise[[3]],
  IMP4=mergelistres_arise[[4]],
  IMP5=mergelistres_arise[[5]],
  IMP6=mergelistres_arise[[6]],
  IMP7=mergelistres_arise[[7]],
  IMP8=mergelistres_arise[[8]],
  IMP9=mergelistres_arise[[9]],
  IMP10=mergelistres_arise[[10]],
  IMP11=mergelistres_arise[[11]],
  IMP12=mergelistres_arise[[12]],
  IMP13=mergelistres_arise[[13]],
  IMP14=mergelistres_arise[[14]],
  IMP15=mergelistres_arise[[15]],
  IMP16=mergelistres_arise[[16]],
  IMP17=mergelistres_arise[[17]],
  IMP18=mergelistres_arise[[18]],
  IMP19=mergelistres_arise[[19]],
```

```
IMP20=mergelistres_arise[[20]],
IMP21=mergelistres_arise[[21]],
IMP22=mergelistres_arise[[22]],
IMP23=mergelistres_arise[[23]],
IMP24=mergelistres_arise[[24]],
IMP25=mergelistres_arise[[25]],
IMP26=mergelistres_arise[[26]],
IMP27=mergelistres_arise[[27]],
IMP28=mergelistres_arise[[28]],
IMP29=mergelistres_arise[[29]],
IMP30=mergelistres_arise[[30]],
IMP31=mergelistres_arise[[31]],
IMP32=mergelistres_arise[[32]],
IMP33=mergelistres_arise[[33]],
IMP34=mergelistres_arise[[34]],
IMP35=mergelistres_arise[[35]],
IMP36=mergelistres_arise[[36]],
IMP37=mergelistres_arise[[37]],
IMP38=mergelistres_arise[[38]],
IMP39=mergelistres_arise[[39]],
IMP40=mergelistres_arise[[40]],
IMP41=mergelistres_arise[[41]],
IMP42=mergelistres_arise[[42]],
IMP43=mergelistres_arise[[43]],
IMP44=mergelistres_arise[[44]],
IMP45=mergelistres_arise[[45]],
IMP46=mergelistres_arise[[46]],
IMP47=mergelistres_arise[[47]],
IMP48=mergelistres_arise[[48]],
IMP49=mergelistres_arise[[49]],
IMP50=mergelistres_arise[[50]],
IMP51=mergelistres_arise[[51]],
IMP52=mergelistres_arise[[52]],
IMP53=mergelistres_arise[[53]],
IMP54=mergelistres_arise[[54]],
IMP55=mergelistres_arise[[55]],
IMP56=mergelistres_arise[[56]],
IMP57=mergelistres_arise[[57]],
IMP58=mergelistres_arise[[58]],
IMP59=mergelistres_arise[[59]],
IMP60=mergelistres_arise[[60]],
IMP61=mergelistres_arise[[61]],
IMP62=mergelistres_arise[[62]],
IMP63=mergelistres_arise[[63]],
IMP64=mergelistres_arise[[64]],
IMP65=mergelistres_arise[[65]],
IMP66=mergelistres_arise[[66]],
IMP67=mergelistres_arise[[67]],
IMP68=mergelistres_arise[[68]],
IMP69=mergelistres_arise[[69]],
IMP70=mergelistres_arise[[70]],
IMP71=mergelistres_arise[[71]],
IMP72=mergelistres_arise[[72]],
IMP73=mergelistres_arise[[73]],
IMP74=mergelistres_arise[[74]],
```

```
  IMP75=mergelistres_arise[[75]],
  IMP76=mergelistres_arise[[76]],
  IMP77=mergelistres_arise[[77]],
  IMP78=mergelistres_arise[[78]],
  IMP79=mergelistres_arise[[79]],
  IMP80=mergelistres_arise[[80]],
  IMP81=mergelistres_arise[[81]],
  IMP82=mergelistres_arise[[82]],
  IMP83=mergelistres_arise[[83]],
  IMP84=mergelistres_arise[[84]],
  IMP85=mergelistres_arise[[85]],
  IMP86=mergelistres_arise[[86]],
  IMP87=mergelistres_arise[[87]],
  IMP88=mergelistres_arise[[88]],
  IMP89=mergelistres_arise[[89]],
  IMP90=mergelistres_arise[[90]],
  IMP91=mergelistres_arise[[91]],
  IMP92=mergelistres_arise[[92]],
  IMP93=mergelistres_arise[[93]],
  IMP94=mergelistres_arise[[94]],
  IMP95=mergelistres_arise[[95]],
  IMP96=mergelistres_arise[[96]],
  IMP97=mergelistres_arise[[97]],
  IMP98=mergelistres_arise[[98]],
  IMP99=mergelistres_arise[[99]],
  IMP100=mergelistres_arise[[100]])

## vector of mediator names, all included in each data frame.
mediators <- c("IIV_arise_p")

## vector of outcome variable names, again all included in each data frame.
outcome <- c("post_isi")

## vector of treatment variables names; must begin with identical strings with dataset
## names in 'datasets'.
treatment <- c("r", "r", "r", "r", "r", "r", "r", "r", "r", "r",
               "r", "r", "r", "r", "r", "r", "r", "r", "r", "r",
               "r", "r", "r", "r", "r", "r", "r", "r", "r", "r",
               "r", "r", "r", "r", "r", "r", "r", "r", "r", "r",
               "r", "r", "r", "r", "r", "r", "r", "r", "r", "r",
               "r", "r", "r", "r", "r", "r", "r", "r", "r", "r",
               "r", "r", "r", "r", "r", "r", "r", "r", "r", "r",
               "r", "r", "r", "r", "r", "r", "r", "r", "r", "r",
               "r", "r", "r", "r", "r", "r", "r", "r", "r", "r",
               "r", "r", "r", "r", "r", "r", "r", "r", "r", "r")

## Set of covariates (in each data set)
covariates <- c("pre_isi + sexnumerisk + age + IIV_arise_b")


x_arise_isi <- mediations(datasets, treatment, mediators, outcome, covariates,
                          families=c("gaussian","gaussian"), interaction=FALSE,
                          conf.level=.95, sims=50)

out_arise_isi <- amelidiate(x_arise_isi)
```

```
## main mediation estimates
summary(out_arise_isi)

plot(out_arise_isi)

## model y and model m
out_arise_isi
```

```
##### HADS
## from whats already defined in the ISI model above, we only have to
## change the outcome and the covariates.

## vector of outcome variable names, again all included in each data frame.
outcome <- c("post_hads_total")

## set of covariates (in each data set), entered using the standard model formula format.
covariates <- c("pre_hads_total + sexnumerisk + age + IIV_arise_b")

x_arise_hads <- mediations(datasets, treatment, mediators, outcome, covariates,
                    families=c("gaussian","gaussian"), interaction=FALSE,
                    conf.level=.95, sims=50)

out_arise_hads <- amelidiate(x_arise_hads)

## main mediation estimates
summary(out_arise_hads)

plot(out_arise_hads)

## model y and model m
out_arise_hads
```
